# Parenteral nutrition: never say never

**DOI:** 10.1186/cc14723

**Published:** 2015-12-18

**Authors:** Taku Oshima, Claude Pichard

**Affiliations:** 1 Clinical Nutrition, Geneva University Hospital, Rue Gabrielle-Perret-Gentil 4, 1211 Geneva 14, Switzerland

## Abstract

This review emphasizes the benefits of parenteral nutrition (PN) in critically ill patients, when prescribed for relevant indications, in adequate quantities, and in due time.

Critically ill patients are at risk of energy deficit during their ICU stay, a condition which leads to unfavorable outcomes, due to hypercatabolism secondary to the stress response and the difficulty to optimize feeding. Indirect calorimetry is recommended to define the energy target, since no single predictive equation accurately estimates energy expenditure. Energy metabolism is intimately associated with protein metabolism. Recent evidence calls for adequate protein provision, but there is no accurate method to estimate the protein requirements, and recommendations are probably suboptimal. Enteral nutrition (EN) is the preferred route of feeding, but gastrointestinal intolerance limits its efficacy and PN allows for full coverage of energy needs.

Seven recent articles concerning PN for critically ill patients were identified and carefully reviewed for the clinical and scientific relevance of their conclusions. One article addressed the unfavorable effects of early PN, although this result should be more correctly regarded as a consequence of glucose load and hypercaloric feeding. The six other articles were either in favor of PN or concluded that there was no difference in the outcome compared with EN. Hypercaloric feeding was not observed in these studies. Hypocaloric feeding led to unfavorable outcomes. This further demonstrates the beneficial effects of an early and adequate feeding with full EN, or in case of failure of EN with exclusive or supplemental PN.

EN is the first choice for critically ill patients, but difficulties providing optimal nutrition through exclusive EN are frequently encountered. In cases of insufficient EN, individualized supplemental PN should be administered to reduce the infection rate and the duration of mechanical ventilation. PN is a safe therapeutic option as long as sufficient attention is given to avoid hypercaloric feeding.

## Introduction

Providing nutrition to critically ill patients has long been challenging, due to the difficulties in determining the nutrition requirements of the patients with heterogeneous characteristics, and selecting the timing and the route of administration. Many investigators have tackled these issues in the form of clinical trials, and guidelines have been created by experts to aid clinicians in making these critical decisions [1,2]. However, these guidelines and recommendations have significantly changed over time due to conflicting and sometimes misleading study results.

The lack of robust evidence has created numerous controversies regarding the best way of providing nutrition in critically ill patients [3]. Enteral nutrition (EN) has been recommended as the first choice of nutritional support for critically ill patients [2,4]. Recently, a few voices have suggested that parenteral nutrition (PN) was potentially dangerous for the critically ill patient [5]. In clinical practice, EN is not always well tolerated due to gastrointestinal intolerance. This leads to a massive energy deficit - that is, the shortfall of energy provision compared with the actual energy needs - by the end of the patient's ICU stay, a condition correlated to poor clinical outcome. This intolerance to EN is of variable amplitude, and if serious becomes an indication for PN which allows for full coverage of energy needs.

Recent studies have focused on this problem and have provided new insights into the practical use of PN. This article aims at drawing the attention of ICU professionals to the benefits of a personalized prescription of PN, which requires optimizing the amount of energy and protein provision, as well as the timing of administration.

## Limitations of enteral nutrition

Although strongly recommended in recent guidelines, optimal nutrition for critically ill patients through the enteral route is nonetheless difficult in some patients because gastrointestinal tolerance limits the rate of administration and absorption. For instance, diarrhea is commonly observed in critically ill patients and is strongly associated with antibiotic treatment, as well as with EN administration if covering more than 60% of the energy needs [6]. Absorption of glucose from EN is impaired as much as three times in critically ill patients [7]. These conditions frequently limit the prescription of full and efficient EN. Nutrients administered by EN must be digested, a process that increases the energy and oxygen demand of the intestine, which in turn increases the splanchnic blood flow [8]. This effect may be poorly tolerated by hemodynamically compromised patients. In such cases, exclusive PN [9] or supplemental parenteral nutrition (SPN) added to a reduced volume of EN for less critical situations may be valuable therapeutic options (Table 1, 2) [10,11].

**Table 1:** Benefits and risks of parenteral and enteral nutrition.

	Parenteral nutrition	Enteral nutrition
Benefits	• Can be administered regardless of gut function	• Preserves gut mucosa and function
	• Composition of formula can be modified according to patient needs	• More physiologic, less invasive
Risks	• Invasive procedure needed for administration	• Gut function limits rate and amount of administration
	• Greater risk of hyperglycemia and overfeeding	• Difficult to modify composition of formula

**Table 2:** Suggested Indications for Parenteral Nutrition.

• Prolonged ileus > 3 days
mechanical obstruction, generalized peritonitis, peritoneal carcinosis, abdominal distension on enteral nutrition
• Short Bowel syndrome
mesenteric infarction, extensive small bowel resection
• Severe malabsorption
radiation injury to intestine, high output fistulae, inflammatory bowel diseases in acute phase, splanchnic ischemia
• Time to reach full enteral nutrition or oral > 5 days
• Insufficient energy intakes
• Hyperemesis gravidarum
• High risk of aspiration

Adapted from [11]

## Parenteral nutrition: the latest studies

In 2014, two large prospective studies challenged the negative opinions against PN (Table 3). Harvey et al. [12] compared the results of administering nutrition either enterally or parenterally. They randomized patients who would have tolerated EN to receive either EN or PN. No significant differences in clinical outcomes (mortality, infection rate) were observed between the two study groups. However, it should be noted that this calories trial was conducted under a local audit, implying a limited external validity. Doig et al. [9] studied patients with relative contraindications to EN, who received PN early or late during the course of their ICU stay. The study design is remarkable because it truly reflects the daily practice. The results were in favor of the early PN strategy with a significant reduction in days on ventilators without an effect on mortality, although the actual difference was limited (-0.047 days per 10 patient-ICU-days).

**Table 3:** Recent studies using parenteral nutrition.

	Early nutrition route		Early PN		SPN		Hypocaloric vs. normocaloric		Optimal protein-energy			Protein nutrition		
	[12]		[9]		[10]		[32]		[33]			[34]		
Study design	Multicenter, pragmatic RT		Multicenter, RCT		Two center, RCT		Prospective RT		Single center, observational study			Single center, observational study		
Study groups	Parenteral	Enteral	Standard care	Early PN	EN group	EN + SPN	Normocaloric	Hypocaloric	No target	Protein-energy	Energy only	Low protein (0.8 g/kg/day)	Medium protein (1.1 g/kg/day)	High protein (1.5 g/kg/day)
Patients enrolled (*n*)	1188	1195	682	681	152	153	54	46	412	245	205	37	38	38
Route of nutrition	PN	EN	EN and/or PN	PN (and EN)	EN	EN + SPN	EN + SPN		EN and/or PN			EN + SPN		
APACHE II score	19.6 ± 6.9	19.6 ± 7.0	21.5 ± 7.8	20.5 ± 7.4	23 ± 7	22 ± 7	27.7 ± 8.4	30.5 ± 8.5	23 ± 8			23.2 ± 7.4	21.9 ± 5.9	22.1 ± 6.8
Energy target	25 kcal/kg BW/day		Not specified	Harris-Benedict equation	Indirect calorimetry, or 25 kcal/kg BW/day (females) and 30 kcal/kg BW/day (males)		Indirect calorimetry, 100% EE, or Ireton-Jones equation	Indirect calorimetry, 50% EE, or Ireton-Jones equation	Harris-Benedict equation with added 20% for stress and 10% for activity until indirect calorimetry; EE from indirect calorimetry + 10% for activity			EE from indirect calorimetry, 25-30 kcal/day before calorimetry		
Protein target	Not specified		Not specified		1.2 g/kg ideal BW		Not specified		1.2-1.5 g/kg preadmission body weight			1.2-1.5 g/kg (classified according to actual provision)		
Primary outcome	No significant difference in death within 30 days between parenteral (33.1%) and enteral (34.2%) groups		No significant difference in crude day-60 mortality (standard care (22.8%) vs. early PN (21.5%))		Significantly reduced late nosocomial infections for EN + SPN (27%) vs. EN (38%)		Significantly higher rate of nosocomial infections for hypocaloric group (26.1%) vs. normocaloric group (11.1%)		50% decrease in 28-day mortality for protein-energy target group compared with no target group			Lower ICU mortality for patients with medium (24%) and high (16%) protein provision compared with low (27%) protein provision		
Secondary outcome	Lower rate of hypoglycemia in parenteral (3.7%) vs. enteral (6.2%); lower rate of vomiting in parenteral (8.4%) vs. enteral (16.2%)		Significantly shorter duration of mechanical ventilation; significantly shorter for early PN		No significant difference in the length of stay in the ICU, the length of stay in the hospital, or mortality		Insulin demand higher in the normocaloric group; no significant difference for blood glucose level, duration of mechanical ventilation, or mortality		No significant difference for meeting energy target alone			APACHE II score, SOFA score, age also predict outcome, amount of energy provision was not related to outcome		

The recent PN studies are shown in the order of their citation in the article. Data are shown as stated in the original article

*APACHE *Acute Physiology and Chronic Health Evaluation, *BW *body weight, *EN *enteral nutrition, *PN *parenteral nutrition, *RCT *randomized controlled trial, *RT *randomized trial, *SOFA *Sequential Organ Failure Assessment, *SPN *supplemental parenteral nutrition

These studies, added to other prospective randomized controlled trials (PRCTs) (Table 3), demonstrate that PN is safe, and should be regarded as an alternative treatment in the case of contraindication or poor tolerance to EN.

## Rationale for prescribing nutrition in ICU patients

Severe stress is a physiological response to critical illnesses, and energy is utilized for a variety of reactions needed for survival. Hypermetabolism is induced by signals from stress hormones, inflammatory cytokines, and other mediators [13]. To meet the energy demand related to hypermetabolism, the body turns to its endogenous energy sources, namely glucose by gluconeogenesis in the liver and free fatty acids by lipolysis in the adipocytes. Plasma amino acids generated from increased skeletal muscle proteolysis also contribute to glucose production in the liver. This process is not reversed by exogenous nutrition whatever the composition, as demonstrated by Tappy et al. [14] who compared the effect of energy provision by isoenergetic PN, either with a glucose-rich formula or a lipid-rich formula. The use of the glucose-rich formula resulted in an elevation of the oxidation of glucose and de novo lipogenesis, while the endogenous glucose production was not changed by the type of formula. Under normal conditions, amino acids in the plasma act as signals to stimulate anabolism of skeletal muscle. In critically ill patients, however, due to the signaling from inflammatory mediators and stress hormones, the anabolic signal of increased plasma amino acids is blunted. Providing proteins or amino acids stimulates protein synthesis to some extent but does not stop catabolism [15]. This phenomenon, termed anabolic resistance, is also a common metabolic alteration observed during the acute phase of critical illness [16,17].

## Nutritional deficits and clinical outcome

Owing to the elevated demand for energy and difficulties in providing adequate nutrition, critically ill patients frequently develop a negative energy balance [18]. In addition, commercial nutrition mixtures are low in protein and critically ill patients are consequently at risk of developing a cumulative deficit of energy and protein during their ICU stay. Such a deficit further aggravates the deleterious impact of critical illness on the lean body mass. Conversely, optimal provision of energy reduces protein catabolism from the skeletal muscle, whereas protein provision primarily improves the rate of protein synthesis in rapidly turning over tissues [19]. Protein-rich nutrition in the early phase of critical illness may be considered a strategy capable of modulating the systemic inflammatory response, while supporting the needed protein synthesis that takes place during this response [20].

Alberda et al. [21] observed in their study that simply increasing nutrition support in the early phase of the ICU stay to minimize the protein-energy deficit improved clinical outcomes. On the other hand, energy deficits have been shown to increase the risk of infectious complications and unfavorable clinical outcomes. Faisy et al. [22] demonstrated that early ICU energy deficit is an independent determinant for acquiring *Staphylococcus aureus *ventilation-associated pneumonia in patients on prolonged mechanical ventilation. This result was supported by Ekpe et al. [23], who showed that methicillin-resistant *S. aureus *(MRSA) bloodstream infections were associated with a higher energy deficit than other ICU-acquired bacteremia. The detrimental effects of undernutrition have been repeatedly reported during the last decades and the recent PRCTs have stimulated experts on both sides of the oceans to recommend the provision of timely and optimal nutrition during critical illness.

## Defining the needs for energy and protein

Current guidelines call for an early initiation of nutrition, but the recommendations for the use of PN are conflicting. The European Society for Clinical Nutrition and Metabolism (ESPEN) guidelines call for EN or PN within 24-48 hours after admission, with the energy provision target as close as possible to the measurement by indirect calorimetry, or 20 kcal/kg/day initially followed by 25 kcal/kg body weight/day after the acute phase when indirect calorimetry is unavailable. The target should be reached within 2-3 days [1]. The American guidelines - that is a joint recommendation by the American Society for Parenteral and Enteral Nutrition (A.S.P.E.N.) and Society of Critical Care Medicine (SCCM) [2] - and the Canadian Critical Care Practice Guidelines (CCPGs) [24] call for an early initiation of EN, but PN should be withheld until 5-7 days [2]. This difference in the recommendations may have significant impact on the early achievement of energy targets, especially when EN is not indicated or poorly tolerated.

**Figure 1:**
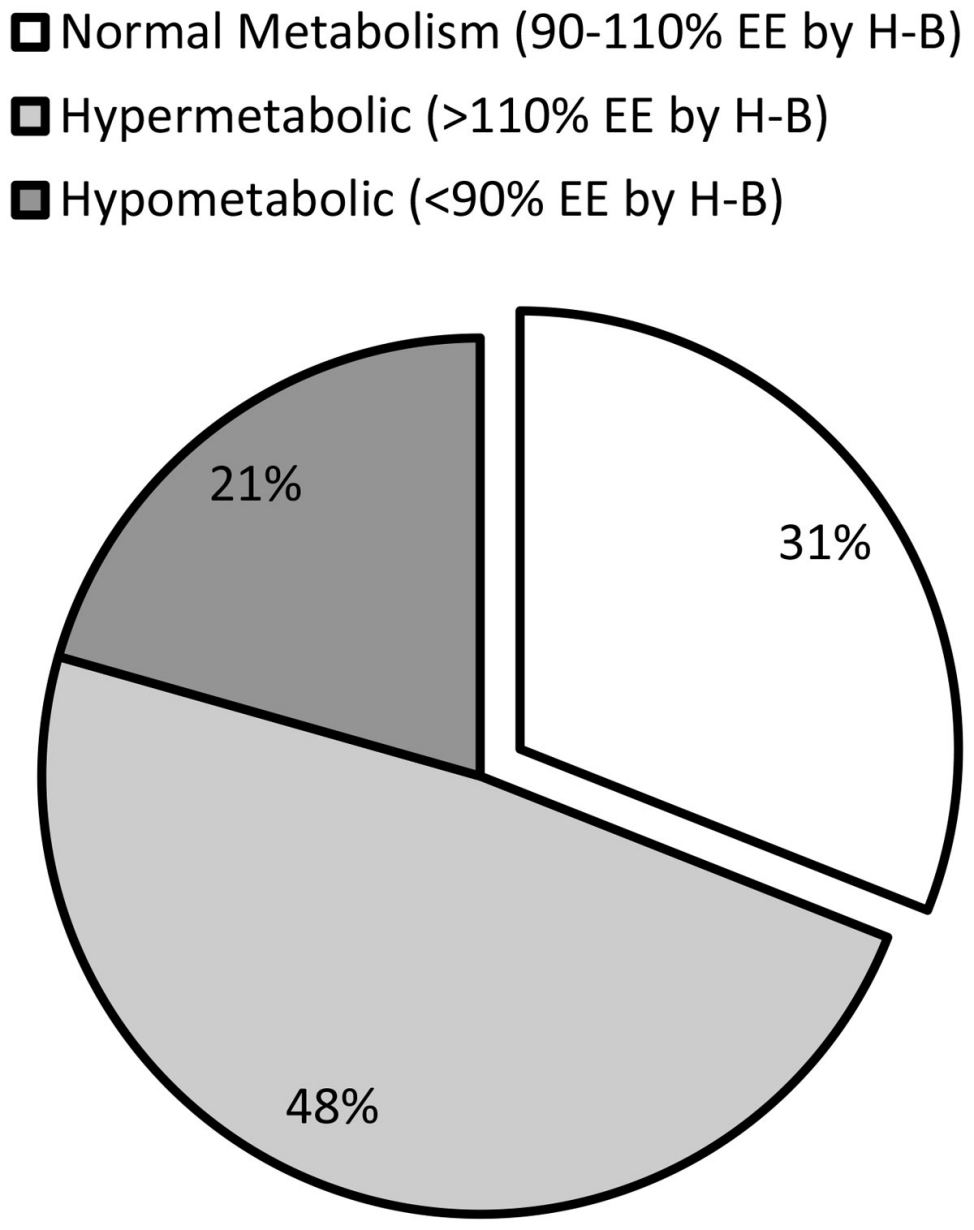
**Distribution of the metabolic state of critically ill patients**. Measured energy expenditure (*EE*) by indirect calorimetry was compared with estimated EE by the Harris-Benedict (*H-B*) equation in 213 critically ill patients. "Normal metabolism" represents those patients with measured EE within 10% of the estimation by the H-B equation. Those patients exceeding 110% and falling short of 90% of the estimated EE were categorized as "hypermetabolic" and "hypometabolic", respectively. Only 31% of the patients had normal metabolism, demonstrating the importance of indirect calorimetry for the accurate assessment of the metabolic state in critically ill patients. Adapted from [27]

Energy targets are usually based on predictive equations. There are over 200 equations to estimate patients' metabolic rates, but only a few are applicable during critical illness [25]. Clinicians turn to these equations largely due to the lack of reliable indirect calorimeters on the market. However, there is no single predictive equation that is accurate during critical illness due to the great heterogeneity of pathologies and treatments [26]. McClave et al. [27] reported that, compared with the measurements by indirect calorimetry, energy expenditure (EE) estimation based on predictive equations was inaccurate for nearly 70% of the critically ill patients (Figure 1). As a result, only about 25% of the patients received an energy provision within 10% of the measured EE [27]. Indirect calorimetry is thus recommended for all critically ill patients for an adequate determination of the energy target.

The importance of protein provision has recently drawn more attention. Unlike energy, there is no specific method to measure the protein need in a timely manner at the bedside. Ishibashi et al. [28] suggested that body protein catabolism over a 10-day period after ICU admission was reduced by 50% when protein intake increased and allowed a reduction of muscle loss of 1.1-1.5 g/kg dry fat-free mass/day. Current trend among experts is to increase intakes up to 1.5-1.8 g/kg/day and consider the protein/energy ratio to optimize the simultaneous amount of protein and energy to be administered. This idea is based on the phenomenon that energy is needed for protein efficacy [29]. Berg et al. [30] compared the whole-body kinetics during hypocaloric feeding (50% EE measured by indirect calorimetry) and full feeding (100% EE) in critically ill patients. Whole-body protein synthesis was lower during hypocaloric feeding while whole-body protein degradation was unaltered, which resulted in a more negative protein balance (-1.9 ± 2.1 vs. -0.7 ± 1.3 mg phenylalanine/kg/hour, *p *= 0.014).

A number of studies have investigated the effects of PN, to achieve energy and protein goals at different timings after ICU admission [31]. Casaer et al. [5] analyzed the impact of an early systematic administration of PN on day 3 after ICU admission, preceded by massive glucose load (400 and 800 kcal during the first 2 days), versus a late initiation of PN on day 8, after 7 days of underfeeding. Tight glycemic control, nowadays shown to be deleterious, was applied to all patients. They found no difference in mortality, but fewer infections, a higher degree of acute inflammation, and shorter duration of mechanical ventilation were observed in the late initiation group. These results were misinterpreted when stating that early PN is harmful for critically ill patients, while relative overfeeding as a result of excessive energy administration by PN in addition to the noninhibitable endogenous energy production is more likely to be the cause of unfavorable outcome. Overfeeding resulted in increased insulin needs to achieve glycemia control in Casaer et al.'s study, in which the patients in the early PN group needed nearly double the amount of insulin for this purpose as shown in the late PN group. These results also emphasize the importance of a precise determination of the energy target, which is only possible with indirect calorimetry.

Heidegger et al. [10] studied the effect of SPN by randomizing patients who were given less than 60% of prescribed energy on the third ICU day to receive either SPN or continue solely on EN (Table 3). Significant reduction (*p *= 0.03) in the rate of late nosocomial infections until 28 days after ICU admission and of antibiotic days per patient, resulting in more antibiotic-free days, was observed in patients who were given SPN to meet energy targets determined by indirect calorimetry, while there were no differences in the mortality or the length of stay in the ICU. Of importance, there was no difference in the number of bloodstream infections in the SPN group versus the EN-only group.

Petros et al. [32] compared critically ill patients who were given nutrition for more than 3 days with energy targets of 50% EE (hypocaloric feeding) and 100% EE (normocaloric feeding). The normocaloric group suffered fewer nosocomial infections (11.6%) compared with the hypocaloric group (26.1%, *p *= 0.046).

Weijs et al. [33] studied mechanically ventilated critically ill patients who were given nutrition according to indirect calorimetry and a protein target of 1.2 g/kg body weight. Optimal provision of both protein and energy was associated with a 50% decrease in 28-day mortality, where only reaching energy targets did not reduce mortality. The results of this study, which addressed the importance of both energy and protein, were further supported by Allingstrup et al. [34]. They demonstrated in an observational study of mixed critically ill patients that only patients receiving a protein delivery >1 g/kg/day had a reduced mortality, regardless of adequate energy delivery. All of these studies support the use of PN for the critically ill patients, provided that they are closely monitored to avoid both hypercaloric and hypocaloric feeding.

## Perspectives

Growing evidence suggests the benefits of providing personalized provision of energy and protein while avoiding both hypocaloric and hypercaloric feeding. To achieve this goal in patients with enteral intolerance or insufficiency, PN under careful monitoring techniques is a relevant and practical solution. Indirect calorimetry is recommended for all critically ill patients, to measure their energy requirements and to monitor metabolic changes during the course of critical illness. Currently, the availability and accuracy of indirect calorimetry is limited [35,36]. An international initiative supported by two European academic societies (European Society for Intensive Care Medicine (ESICM) and ESPEN) is presently developing an accurate, easy-to-use, and affordable calorimeter to promote a wider use of this technique.

Methods to measure the protein requirements and to correctly assess the result of protein and amino acid provision are needed for the better fine-tuning of protein provision. There are also new possibilities for the use of PN. Pradelli et al. [37] showed in a large multisite study the benefits of providing immune-modulating lipids using PN. This and other new ideas that may arise must be studied with careful scientific and clinical logic to avoid misinterpreted and misleading ideas from interfering with the progress of patient care.

## Conclusion

PN has been regarded as harmful to critically ill patients, although this conception was not necessarily based on appropriate scientific or clinical logic. The current recommendation of nutrition for the critically ill patients is first to avoid both hypocaloric and hypercaloric feeding. The same attention should also be given to the adequate provision of protein. EN is the first choice for critically ill patients, but it is frequent to encounter difficulties providing optimal nutrition through exclusive EN. In the case of insufficient EN, individualized supplemental PN should be administered to reduce the infection rate and the duration of mechanical ventilation. PN is a safe therapeutic option as long as sufficient attention is given to avoid hypercaloric feeding (Table 4).

**Table 4:** Key messages about the clinical use of enteral and parenteral nutrition in ICU patients.

Conclusion	
• Both hypocaloric and hypercaloric feeding are unsafe	
• Enteral nutrition: preferred route of nutrition	
• Start enteral nutrition on day 1 or 2 after ICU admission	
• In case of failure with enteral nutrition on day 3 or 4 after ICU admission, start individualized supplemental parenteral nutrition to reduce infection rates and duration of mechanical ventilation	
• Parenteral nutrition is safe as long as hypercaloric feeding is avoided	
• Exclusive parenteral nutrition should be reserved to absolute contraindication to enteral nutrition	

## Abbreviations

EE, Energy expenditure; EN, Enteral nutrition; PRCT, prospective randomized controlled trials ESPEN, European Society for Clinical Nutrition and Metabolism; MRSA, Methicillin-resistant *Staphylococcal aureus*; PN, A.S.P.E.N., American Society for Parenteral and Enteral Nutrition; Parenteral nutrition; SCCM, Society of Critical Care Medicine; CCPGs, Canadian Critical Care Practice Guidelines; ESICM, European Society for Intensive Care Medicine SPN, Supplemental parenteral nutrition.

## Competing interests

CP received financial support as research grants and an unrestricted academic research grant, as well as a nonrestrictive research grant and consulting fees, from Abbott, Baxter, B. Braun, Cosmed, Fresenius-Kabi, Nestle Medical Nutrition, Novartis, Nutricia--Numico, Pfizer, and Solvay, outside the submitted work. TO received financial support as an unrestricted academic research grant from public institutions (Geneva University Hospital) and the Foundation Nutrition 2000 Plus.
